# Opportunities for Quality Improvement Programs (QIPs) in the Nutrition Support of Patients with Cancer

**DOI:** 10.3390/healthcare8030227

**Published:** 2020-07-24

**Authors:** Mary Beth Arensberg, Julie Richards, Jyoti Benjamin, Kirk Kerr, Refaat Hegazi

**Affiliations:** 1 Abbott Nutrition Division of Abbott, Columbus, OH 43219, USA; kirk.kerr@abbott.com (K.K.); refaat.hegazi@abbott.com (R.H.); 2 Nutrition Consultant, Columbus, OH 43212, USA; julierichards.rdn@outlook.com; 3 Factoria Medical Center, Kaiser, Permanente, Bellevue, WA 98006, USA; jbenjamin98@hotmail.com

**Keywords:** malnutrition, ambulatory cancer care, quality improvement programs, malnutrition screening, nutrition interventions, quality care, health outcomes

## Abstract

Malnutrition in patients with cancer is a ubiquitous but neglected problem that can reduce patient survival/quality of life and increase treatment interruptions, readmission rates, and healthcare costs. Malnutrition interventions, including nutrition support through dietary counseling, diet fortification, oral nutrition supplements (ONS), and enteral and parenteral nutrition can help improve health outcomes. However, nutritional care standards and interventions for cancer are ambiguous and inconsistently applied. The lack of systematic malnutrition screening and intervention in ambulatory cancer care has especially significant consequences and thus the nutrition support of patients with cancer represents an area for quality improvement. United States healthcare payment models such as the Oncology Care Model are linked to quality of care and health outcomes. Quality improvement programs (QIPs) can advance patient-centered care, perfect care processes, and help healthcare professionals meet their quality measure performance goals. Malnutrition QIPs like the Malnutrition Quality Improvement Initiative (MQii) have been shown to be effective in identifying and treating malnutrition. However, little is known about or has been reported on nutrition or malnutrition-focused QIPs in cancer care. This paper provides information to support translational research on quality improvement and outlines the gaps and potential opportunities for QIPs in the nutrition support of patients with cancer.

## 1. Introduction

Malnutrition in patients with cancer is a ubiquitous but neglected problem that continues to remain the “elephant in the room” [1]. Indeed, up to 80% of patients with solid tumors develop malnutrition—most often protein-energy undernutrition—during the course of their cancer care [2,3,4]. Malnutrition negatively impacts many health outcomes. Two decades ago, it was identified that the loss of even 5% of body weight decreased survival in patients with cancer [5], and multiple studies have since corroborated the association between weight loss and poor cancer outcomes [6,7,8]. The Evidence Analysis Library of the Academy of Nutrition and Dietetics (Academy) has documented strong evidence (Grade 1) of an association between poor nutrition status in adult oncology patients and decreased tolerance to radiation treatment, decreased tolerance to chemotherapy treatment, increased length of hospital stay, lower quality of life, and mortality [9]. Malnutrition in patients with cancer is also associated with higher healthcare costs [10].

Malnutrition interventions, including nutrition support through dietary counseling, diet fortification, oral nutrition supplements (ONS), and enteral and parenteral nutrition, can help improve health outcomes [11]. However, in the American outpatient setting, where nearly all oncology patients receive treatment, ambulatory nutritional care standards and interventions for cancer are ambiguous and inconsistently applied [10]. This is further complicated by the fact that registered dietitian nutritionists (RDNs) are not routinely employed in outpatient cancer centers and the medical nutritional therapy that they provide is not consistently a part of multidisciplinary outpatient cancer care or adequately reimbursed [12]. The lack of systematic malnutrition screening and intervention in ambulatory cancer care has especially significant consequences for both patient and healthcare outcomes and thus the nutrition support of patients with cancer represents an important area for quality improvement.

In the United States (U.S.), healthcare payment models are increasingly linked to quality of care and health outcomes. For example, the U.S. Oncology Care Model (OCM) is a specialty payment program that aims to provide greater quality, more highly coordinated cancer care at the same or lower cost. Physician practices participating in the OCM commit to providing enhanced services to their Medicare patients. Such services can include care coordination, navigation, and aligning with national treatment guidelines [13]. Systematic malnutrition screening and intervention are not required but are a good fit with these enhanced services, particularly because malnutrition impacts health outcomes. The OCM also includes performance against specific quality measures, including unnecessary Emergency Department (ED) visits and patient satisfaction, as well as total cost of care thresholds. Poor health outcomes related to malnutrition, such as hospital readmissions and ED visits, can reduce physicians’ Medicare payments.

As improving quality of care continues to gain momentum in all care segments, healthcare professionals are using quality improvement programs (QIPs) to advance patient-centered care, perfect care processes, and meet their quality measure performance goals. Malnutrition QIPs have been shown to be effective in identifying and treating malnutrition [14]. However, little is known about or has been reported on nutrition or malnutrition-focused QIPs in cancer care, even though the Oncology Nutrition Dietetic Practice Group of the Academy has identified quality improvement as one area where more translational research is needed [10].

This paper provides information to support translational research on quality improvement and the development of malnutrition-focused QIPs for cancer care. First, this paper briefly reviews the healthcare quality improvement process, including the Malnutrition Quality Improvement Initiative (MQii). Next, the paper reports on various quality frameworks for nutrition in adult cancer care, identifying international and U.S. nutrition-specific oncology care guidelines as well as the presence or absence of nutrition in general U.S. cancer care guidelines, standards, quality measures, and initiatives. Finally, the paper describes the evidence for malnutrition and nutrition QIPs in cancer care. In summary, this review—intended for individual teams and healthcare organizations—outlines the gaps and potential opportunities for QIPs in the nutrition support of patients with cancer.

## 2. The Healthcare Quality Improvement Process

**Figure 1:**
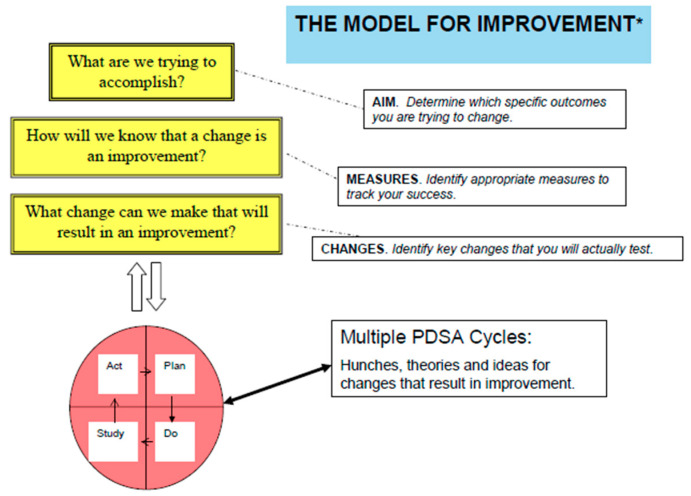
The Model for Improvement [16] used in healthcare quality improvement. * The Plan Do Study Act (PDSA) cycle was developed by W. Edwards Deming [17].

The healthcare quality improvement process can seem overwhelming, but it does not have to be. Healthcare quality improvement is patient-focused and thus the goal of a QIP is to identify how care processes can be improved to benefit patient health outcomes. Ideally, the process starts with a single care team or small unit of caregivers, such as a cancer care or nutrition support team, and then often evolves to include behavior and practice changes across multiple levels of a healthcare organization. QIP models vary in their approach and methods; however, all reflect the common principle that quality improvement is a continuous activity, not a single event. Thus, as changes are implemented, ongoing issues are addressed, and further changes are made to perfect the targeted patient care process. In healthcare, the most common QIP is the Model for Improvement (MFI), which uses a rapid cycle process called Plan Do Study Act (PDSA) (Figure 1) [15].

To begin the PDSA cycle, the team

1. Establishes improvement goals
2. Identifies possible strategies
3. Chooses specific interventions to implement
4. Prepares a written action plan.

Through successive PDSA cycles, clinicians arrive at a final process change which they believe is most effective in producing the desired results. Subsequently, they may work to implement and spread this process change through their broader healthcare organization. Ultimately, it is the small changes perfected through the PDSA cycle which provide an opportunity for larger and more lasting effects on the healthcare system’s quality of patient care [15].

**Figure 2:**
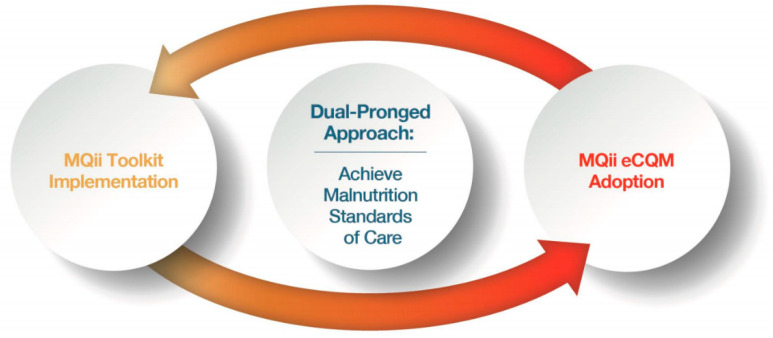
The Malnutrition Quality Improvement Initiative (MQii) dual-pronged approach to helping hospitals achieve malnutrition standards of care [14].

The Malnutrition Quality Improvement Initiative (MQii) is a malnutrition-specific QIP framework using the PDSA cycle that could serve as a model for nutrition-focused QIPs in cancer care. The MQii began in 2013, when a variety of stakeholder organizations highlighted gaps in existing malnutrition care and the impact of these gaps on patient outcomes. Following literature reviews, landscape assessments, engagements with key opinion leaders, and best practice research, the MQii was established in partnership with the Academy, Avalere Health, and other stakeholders. Support for the MQii has been provided by Abbott. MQii innovations include the development of an evidence-based malnutrition quality improvement toolkit and a set of malnutrition electronic clinical quality measures (eCQMs) (Figure 2) [14]. The MQii toolkit is interdisciplinary and open-access [18]; further details on the four malnutrition eCQMs are available on the Academy’s website [19].

The MQii initially focused on advancing evidence-based, high-quality, patient-centered care for hospitalized older adults (aged 65 years and older). To help healthcare institutions achieve malnutrition standards of care, the MQii also established a learning collaborative, which currently boasts a membership of over 250 U.S. healthcare institutions. Results from the learning collaborative demonstrated that hospital teams which implemented the MQii improved the timely identification, quality of care, and treatment of older adults who were malnourished or at risk of malnutrition. RDNs and their interdisciplinary colleagues have led this implementation process. Furthermore, the MQii is helping to expand learning collaborative hospitals’ leadership into transitions of care, securing nutrition quality measures in core data sets and national data registries, and implementing the MQii model in other care settings and with other patient populations [20]. Specific applications of the MQii model to cancer care alone have not yet been reported.

## 3. Quality Frameworks for Nutrition in Cancer Care

QIPs such as the MQii often focus on improving patient care processes by more closely aligning the processes with recommended clinical guidelines, standards, quality measures, best practices, and other quality frameworks. For example, one way in which physician practices participating in the OCM can enhance their services is by following national treatment guidelines. However, in nutrition-specific oncology care, the standards and guidelines are inconsistent and may be of limited quality [21]. Furthermore, nutrition is frequently not included in general oncology standards, guidelines, quality measures, and initiatives. The following summarizes existing nutrition-specific oncology care quality frameworks and describes where nutrition is or is not included in the quality frameworks of general oncology care. The gaps may be helpful for individual teams to identify possible areas for malnutrition QIPs and for healthcare organizations to identify opportunities for improvement in national cancer care guidelines, standards, and quality programs.

### 3.1. International Nutrition-Specific Oncology Care Guidelines

A recent international review of nutrition care procedures in nutrition-specific guidelines for patients with cancer identified 17 guidelines [21]. Using the Appraisal of Guidelines for Research and Evaluation (AGREE II) methodology to evaluate the quality of those guidelines, Zhao et al. rated the European Society for Clinical Nutrition and Metabolism (ESPEN) [11] guidelines and Australian guidelines [22] as having the highest total quality scores (>60%). They further evaluated 12 of the guidelines and found heterogeneity in the content/tools of nutrition screening and/or assessment, application of immune nutrients, and selection of nutrition support pathways, and they concluded that the quality of nutrition guidelines for patients with cancer was highly variable [21].

### 3.2. U.S. Nutrition-Specific Oncology Care Standards and Guidelines

Of the 17 nutrition-specific oncology care guidelines evaluated by Zhao et al., only two were from the U.S. [9,23]. However, in broadening the approach to include nutrition-specific standards of practice, several additional U.S. nutrition-specific quality frameworks for cancer care can be identified (Table 1).

### 3.3. Nutrition in U.S. General Oncology Care Standards and Guidelines

Nutrition is lacking in many of the U.S. general oncology care standards and guidelines. However, specific sections on nutrition are part of the American College of Surgeons (ACS) Commission on Cancer (CoC) Accreditation Standards, the Association of Community Cancer Centers (ACCC) Cancer Program Guidelines, several Enhanced Recovery after Surgery (ERAS^®^) Society guidelines, and in the National Cancer Institute’s Physicians Data Query^®^ resources (Table 2).

The National Comprehensive Cancer Network (NCCN) is an alliance of 28 leading U.S. cancer centers focused on improving the quality of cancer care. NCCN guidelines are evidence-based, consensus-driven, and reported to be the most thorough and frequently updated clinical practice guidelines in any area of medicine [32], but most contain limited if any nutrition recommendations or information (Table 3 and Supplementary Materials). Not surprisingly, the diagnoses with more frequent nutrition mentions in the NCCN guidelines all involve some part of the gastrointestinal tract. The most common references to nutrition are related to nutrition status, nutrition counseling, and nutrition support. Further details of the frequencies and types of references to nutrition in the NCCN guidelines are provided in the appended Supplementary Materials.

Compared to NCCN guidelines, the American Society of Clinical Oncology (ASCO) guidelines have even fewer mentions of nutrition. One new ASCO Management of Cancer Cachexia guideline has a significant focus on nutrition. However, of the other more than 90 ASCO guidelines, nutrition is mentioned in just two guidelines; once in the Management of Osteoporosis in Survivors of Adult Cancers with Nonmetastatic Disease guideline and twice in the Practical Assessment and Management of Vulnerabilities in Older Patients Receiving Chemotherapy guideline [33]. In summary, few U.S. general oncology care standards and guidelines mention nutrition and even fewer include it as a specific area of focus.

### 3.4. Nutrition in U.S. Cancer Care Quality Measures, Initiatives, and Data Sources

Nutrition is nearly absent in U.S. cancer care quality measures and initiatives. In the oncology quality care measures developed by national organizations, specifically ASCO’s more than 150 quality measures [34], CoC’s 23 quality measures [35], the National Quality Forum (NQF)’s 16 quality measures [36], and the Oncology Nursing Society’s 10 quality measures [37], no quality measures are specific to or mention nutrition. Furthermore, nutrition is not part of any of ASCO’s cancer care quality initiatives [38], such as the Quality Training Program (QTP), Quality Oncology Practice Initiative (QOPI), and Quality Certification Program (QCP), and there is just one project specific to nutrition in the more than 80 QTP projects listed in the ASCO Quality Improvement Library. Additionally, the American College of Surgeon and American Cancer Society’s National Cancer Database [39], a hospital data registry used for quality benchmarking, does not include nutrition as a variable in the publicly available data.

## 4. Review of Malnutrition and Nutrition-Focused QIPs in Cancer Care

To review the evidence base on malnutrition and nutrition-focused QIPs in cancer care, we conducted a search in Embase^®^ and Medline^®^ on research from developed countries published in English between 1 January 2000 to 31 December 2019. Our search was exclusive to research in the adult population (aged 18 years and older), in inpatient and outpatient settings, and for all active cancer diagnoses (we excluded patients in hospice/palliative care). The search terms are detailed in Table 4. Key words were linked using “OR” as a Boolean function and the results of the components were combined using the “AND” Boolean function. All study designs were included and duplicate studies were removed from the final abstract count. A total of 228 abstracts were identified and then screened independently by two reviewers to distinguish which publications were specific to malnutrition and nutrition-focused QIPs or quality effectiveness process initiatives in cancer care. The two reviewers then met and agreed that seven abstracts satisfied the criteria. Of these, only one was a peer-reviewed article; the remaining six were conference abstracts.

Two conference abstracts summarized specific quality improvement programs; both aimed to increase the rate of RDN-documented nutrition assessment and plan of care through successive PDSA cycles. Brown et al. had a goal of increasing the rate of an RDN-documented nutrition assessment to 65%; at baseline, 41.1% of new patients had RDN-documented nutrition plans within 90 days of their first appointment. Multiple causes of the low nutrition plan baseline rates were identified, including those related to patient or family characteristics/needs, clinical dietitian resources, physician limitations, process flaws, and difficulty with the electronic medical record [40]. Levonvak et al. reported that the rate of a documented nutrition care plan doubled after a month of starting the second PDSA cycle intervention [41].

The other five abstracts described the evaluation of the effectiveness of new nutrition-related clinical processes, pathways, models of care, and/or roles [42,43,44,45,46]. One conference abstract and the one article evaluated the benefit of training a nutrition assistant to perform nutrition screening and intervention for oncology patients and found that the role of nutrition assistants could benefit patient outcomes [42,46]. Further information about the seven abstracts is summarized in Table 5.

## 5. Discussion

Malnutrition has long been linked to poor patient and health outcomes, including reducing patient survival and quality of life and increasing treatment interruptions, readmission rates, and healthcare costs. Evidence indicates that early nutrition intervention can reduce complication rates, lengths of hospital stay, readmission rates, mortality, and costs of care [10]. However, for many patients, malnutrition continues to go unrecognized and untreated [47]. Similarly, while nutrition support is recognized as critical for oncology care, and recent evidence continues to show that specific interventions such as enteral and parenteral feeding are associated with positive survival benefits in patients with metastatic disease [48], malnutrition remains a frequent comorbid condition for patients with cancer [49]. During the last decade, the collaborative and visionary leadership of the MQii and the implementation of malnutrition and nutrition-focused QIPs nationwide have helped to advance malnutrition care for hospitalized patients in the U.S. Unfortunately, there has been less progress in malnutrition care in oncology and at present there are few reported models of well-developed U.S. patient care programs providing optimal malnutrition care. There may be several reasons for this gap in care and thus potential opportunities for individual teams and healthcare organizations to advance malnutrition care in the nutrition support of patients with cancer.

Firstly, there are no quality models specific to malnutrition or nutrition care in oncology that provide a framework for comprehensive care. The MQii was initially developed to target hospital patients and older adults specifically. Only recently has there been a focus by RDNs and other healthcare professionals to promote MQii learnings across the continuum of care and with different patient populations [20]. One U.S. teaching hospital has reported using elements of the MQii Toolkit to measure and improve quality, and as a part of their initiative, they began providing free, early immunonutrition supplements and nutrition education to high-risk patients with colorectal cancer during preadmission testing. Overall, they reported significant reductions in length of stay (LOS) (from 8 to 6 days, *p* < 0.01) and infection rates (from 14% to 9%, *p* < 0.01) for patients who were malnourished or at risk of malnutrition, although they did not report specific results for the cancer subpopulation [50]. Similar to this institution, cancer care teams could readily adapt MQii tools and processes for use in oncology clinics to provide more inclusive care. The broader implementation of ERAS^®^ is also an opportunity to model quality malnutrition care and impact outcomes. ERAS^®^ is not a single, rigid protocol but rather a comprehensive new way for multidisciplinary teamwork to make changes as knowledge evolves [51]. ERAS^®^ includes a significant nutrition component and ERAS^®^ has been shown to decrease complications, reduce LOS, and save costs for oncology patients undergoing surgery [52,53,54]. Another recommendation to incentivize more comprehensive care is for healthcare organizations to advocate for the addition of malnutrition care to future updates of the OCM. The Academy has recently strongly encouraged the inclusion of medical nutrition therapy into the care design and payment for CMS’ next proposed payment model—the Oncology Care First model. It reinforced that “there is strong (grade I) evidence for evaluation of nutritional status as a key component of the oncology patient care process” [55].

Secondly, while there are several nutrition-specific oncology care guidelines, they are inconsistent and nutrition is usually not included in general oncology care standards and guidelines; in contrast to other countries, U.S. oncology care guidelines do not recommend frequent interaction or access to oncology nutrition services [56]. This can make it more difficult to initiate nutrition-focused QIPs to optimize patient and health outcomes. U.S. oncology care guidelines could benefit from education and awareness building, starting with medical education, although changing guidelines is a lengthy process. There are examples of nutrition support for patients with cancer included as a part of medical school nutrition curriculum [57] but, unfortunately, the adoption of such nutrition curriculum has not been widespread. In a recent systematic review, Crowley et al. identified insufficient inclusion of nutrition in medical education, regardless of country, setting, or year of medical education [58]. Walsh et al. noted that only 25% of medical schools have a dedicated nutrition curriculum and few meet the National Academy of Science’s recommended 25 h of nutrition education. Furthermore, Walsh et al. have pointed out that the American Board of Internal Medicine’s Maintenance of Certification for Medical Oncology Fellows does not have any requirements related to nutrition [1]. Malnutrition advocates have purposely targeted the inclusion of nutrition in medical standards and guidelines. For example, the Malnutrition Quality Collaborative in their *National Blueprint: Achieving Malnutrition Care in Older Adults, 2020 Update* makes the specific recommendation to “Develop core materials and integrate malnutrition care training modules into school and university curriculums for physicians” [59]. Another approach could be to engage clinicians directly. For example, the Prevalence of Malnutrition in Oncology (PreMiO) study was conducted at 22 medical oncology centers across Italy to raise oncologists’ awareness of the “pressing need for early assessment of nutrition status in cancer patients and the need for providing appropriate nutritional care.” In the model, oncologists—rather than RDNs—evaluated the nutrition status of patients at their first medical oncology visit and results demonstrated that oncologists could be effectively trained to perform assessments that identified malnutrition and its risks [3]. The role of the oncologist in interdisciplinary nutrition care is further delineated in a review by Muscaritoli et al., where they outline a roadmap for oncologists to move from guidelines to clinical practice [60]. To date, no such initiatives seem to have been undertaken in the U.S., although the need to develop a “culture of nutrition” among all cancer care staff has been identified [56]. Cancer care teams can help bring precedence to the need for including nutrition in general oncology recommendations and standards by implementing and publishing the results of malnutrition and nutrition-focused QIPs.

Thirdly, based on the evaluation of NCCN guidelines, patient recommendations have limited focus on nutrition. Mentions of nutrition in the NCCN guidelines are more frequent in the clinical diagnosis-specific guidelines compared to the patient guidelines. This is of concern because, at its core, healthcare quality improvement and comprehensive cancer care are patient-focused. Rauh et al. have stated that “Nutrition is a major issue for most patients with cancer and their families, and its impact will often lead to highly emotionalised discussions in our daily practice. For all participants, there often is an unpronounced underlying fear: that their cancer may already have ‘consumed’ the patient, and thus ‘won’. On the other hand, nutrition is one factor they potentially (think they) can influence.” Rauh et al. go on to describe the Cancer Patient’s Nutritional Bill of Rights, which was based on ESPEN’s guidelines for nutrition in cancer patients, published by the European Cancer Patient Coalition, and presented to the European Parliament in November 2017 [61]. Cancer care teams and healthcare organizations could consider creating and adopting a similar Bill of Rights in the U.S. to empower patients, their families, and healthcare providers to systematically identify and intervene for malnutrition in cancer care which could help improve both patient and healthcare system outcomes.

Fourthly, there is a complete lack of U.S. nutrition quality measures for oncology. In the over 200 distinct quality measures for cancer care identified, none were specific to nutrition. The Academy, in describing their first ever malnutrition eCQMs, recognized the availability of these eCQMs as a “tremendous opportunity to advance patient/client nutrition care.” They further underscored that “the development of the eCQMs and tools to support implementation is one of the most innovative initiatives undertaken by the Academy of Nutrition and Dietetics” [62]. Consistent malnutrition screening is critical for the early identification and treatment of malnutrition; however, in a survey of U.S. ambulatory oncology settings, only about half reported screening for malnutrition [12]. Targeting the development of malnutrition quality measures specific to cancer care should be a priority for nutrition and oncology organizations because eCQMs are an effective tool to help identify where malnutrition screening is not occurring as well as to document the burden of malnutrition and the positive outcomes that are realized when malnutrition is better identified and treated.

Lastly, there is a void of malnutrition and nutrition-focused QIPs and data sources for nutrition support in cancer care. In our review, we found very few published QIPs for malnutrition and nutrition care in oncology and all but one were conference abstracts vs. peer-reviewed articles. Published QIPs can help provide examples for other clinicians to mirror, particularly as there are few published best practice models in the U.S. of oncology-focused patient care programs to prevent and treat malnutrition. The *Journal of the Academy of Nutrition and Dietetics* recently published a special supplement on the MQii to “provide a guide or template for individuals and organizations interested in continually improving the nutrition care in their respective facility regardless of their particular situations and resources” [14]. There are also nutrition-focused QIPs where the study population includes patients with cancer. In one such study, oncology patients using ONS as part of the QIP were associated with 46.1% fewer 30-day hospital readmissions after controlling for other covariates and confounding variables (*p* < 0.001) [63]. Similarly, a secondary analysis of at-risk or malnourished hospital patients with cancer in a nutrition-focused QIP documented statistically significant reductions in 30-day hospital readmission rates and lengths of stay, with potential cost savings of >$3800 per patient treated [64]. As clinicians develop and implement malnutrition and nutrition-focused QIPs, they should work to publish their results and include the QIPs in ASCO’s Quality Improvement Library to help increase visibility among interdisciplinary care teams nationwide. There is also a need for benchmarking data that can be used for quality care comparisons for oncology malnutrition care. Thus, it is recommended that healthcare organizations consider how data sources, such as the National Cancer Database, can be expanded to include specific data points relevant to malnutrition and nutrition care.

## 6. Conclusions

Existing literature shows that poor nutrition remains a challenge for both patients with cancer and healthcare providers, adversely impacting morbidity and mortality, decreasing quality of life, and driving substantial cost burdens for the healthcare system. The lack of emphasis on nutrition in oncology treatment guidelines in the U.S. suggests that nutrition is an under-utilized tool in cancer treatment. The nature of much of cancer care taking place in outpatient clinics makes this an ideal setting for the successful execution of a QIP. Specifically, patients have regular office visits for treatment and follow-up, and, during these visits, their nutrition status and risk for malnutrition could be monitored, recommendations made, and compliance verified. Such a coordinated effort would require the involvement of multiple patient care disciplines but could yield significant improvements in patient outcomes and quality of life. There is an opportunity for individual teams and healthcare organizations to help fill such existing gaps in malnutrition care in oncology clinics and, as documented in this paper, implement and then leverage QIPs focused on improving the nutrition support of patients with cancer.

## Figures and Tables

**Table 1:** U.S. nutrition-specific oncology care standards and guidelines.

Organization and Developer	Title and Target Audience	Methodology and Scope
Academy of Nutrition and Dietetics (the Academy) Oncology Workgroup	Oncology Evidence-Based Nutrition Practice Guideline for Adults [9] Registered Dietitian Nutritionists (RDNs)	Systematic review of research published 1990–2013 Focuses on: Validity of malnutrition screening, nutrition assessment tools ○ Association among nutrition status and morbidity/mortality outcomes ○ Effect of medical nutrition therapy (MNT) on patients undergoing cancer therapies, cancer cachexia ○ Effect of dietary supplements and medical food supplements with fish oil on body weight/lean body mass
Academy Oncology Nutrition Dietetic Practice Group, with guidance from Academy Quality Management Committee	Academy of Nutrition and Dietetics: Revised 2017 Standards of Practice and Standards of Professional Performance for Registered Dietitian Nutritionists (Competent, Proficient, and Expert) in Oncology Nutrition [24] RDNs	Address current skill level, identify areas for additional professional development Address/apply nutrition care process and workflow elements: ○ screening, assessment, diagnosis, intervention, evaluation/monitoring, discharge planning, transitions of care Include six domains of professionalism: ○ Quality in practice; competence and accountability; provision of services; application of research; communication and application of knowledge; utilization and management of resources
American Society for Parenteral and Enteral Nutrition (ASPEN) ASPEN Members	Review of American Society for Parenteral and Enteral Nutrition (ASPEN) Clinical Guidelines for Nutrition Support in Cancer Patients: Nutrition Screening and Assessment [25] Multidisciplinary teams	Update 2002 ASPEN Clinical Guidelines Provide background on nutrition in cancer patients Discuss role of nutrition screening and assessment in cancer care
ASPEN ASPEN Members	Nutrition Support in Surgical Oncology [26] Multidisciplinary teams	Update 2002 ASPEN Clinical Guidelines Evaluate evidence related to use of nutrition support in surgical oncology patients
ASPEN ASPEN Members and Board of Directors	ASPEN Clinical Guidelines: Nutrition Support Therapy During Adult Anticancer Treatment and in Hematopoietic Cell Transplantation [23] Multidisciplinary teams	Update 2002 ASPEN Clinical Guidelines Created in accordance with Institute of Medicine recommendations

**Table 2:** U.S. general oncology care standards and guidelines including nutrition.

Organization	Reference	Methodology and Scope
American College of Surgeons (ACS) ACS Multidisciplinary Commission on Cancer	Commission on Cancer. Optimal Resources for Cancer Care (2020 Standards) [27] Accredited U.S. cancer programs	Developed to ensure quality, multidisciplinary, comprehensive cancer care delivery in healthcare settings Specified oncology nutrition services components: ○ Screening/assessment for risk/diagnosis of malnutrition, nutrition-related problems, overweight/obesity ○ Medical nutrition therapy ○ Nutrition counseling/education ○ Management/coordination of enteral and parenteral nutrition Annual compliance measure for oncology nutrition services: ○ Oncology nutrition services provided, on-site or by referral by RDN ○ Process monitored/reviewed/documented by accredited institution’s cancer committee
Enhanced Recovery After Surgery (ERAS^®^) Society Joint efforts of the ERAS^®^ Society and authors from the international ERAS^®^ Gynecology chapters	Guidelines for Perioperative Care in Gynecologic/Oncology: Enhanced Recovery After Surgery (ERAS^®^) Society Recommendations-2019 Update [28] Surgical teams	Recommendations based on grading of recommendations, assessment, development, and evaluation (GRADE) Present updated consensus review of perioperative care for gynecologic/oncology surgery based on best current evidence
ERAS^®^ Society Endorsed by ERAS^®^ Society and international panel of experts in major head/neck cancer surgery and enhanced recovery after surgery	Optimal Perioperative Care in Major Head and Neck Cancer Surgery with Free Flap Reconstruction, a Consensus Review and Recommendations from the Enhanced Recovery After Surgery Society [29] Surgical teams	Recommendations based on grading of recommendations, assessment, development and evaluation (GRADE) Systematic review and expert evaluation to provide consensus-based protocol for optimal perioperative care of patients undergoing head and neck cancer surgery with free flap reconstruction Evidence base for specific perioperative care elements in head and neck cancer surgery is variable; in many cases, information from different surgical procedures forms basis for these recommendations
ERAS^®^ Society ERAS^®^ Society working group	Guidelines for Perioperative Care after Radical Cystectomy for Bladder Cancer: Enhanced Recovery After Surgery (ERAS^®^) Society recommendations [30] Surgical teams	Systematic review to analyze application of ERAS^®^ protocols and evidence for individual ERAS^®^ items for cystectomy Provide comprehensive ERAS^®^ pathway for cystectomy based on available evidence and assimilating recommendations for other pelvic surgeries where appropriate
National Cancer Institute PDQ^®^ Supportive and Palliative Care Editorial Board	PDQ^®^ Nutrition in Cancer Care [31] Clinicians	Uses formal evidence ranking system in developing level-of-evidence designations Comprehensive, peer-reviewed, evidence-based information about nutrition before, during, and after cancer care Does not provide formal guidelines/recommendations for making healthcare decisions

**Table 3:** Summary of nutrition mentions in adult National Comprehensive Cancer Network (NCCN) guidelines [32] *.

Type of NCCN Guideline (*N*)	Number (%) of NCCN Guidelines with Nutrition Mentions	NCCN Guidelines with Specific Nutrition Section
Diagnosis-specific guidelines (53)	19 (36%) 1–10 nutrition mentions 3 (6%) 11–25 nutrition mentions 3 (6%) >25 nutrition mentions	Head and neck cancer
Population-specific guidelines (2)	1 (50%) 1–10 nutrition mentions 1 (50%) >25 nutrition mentions	Older adult oncology
Supportive care guidelines (12)	6 (50%) 1–10 nutrition mentions 3 (25%) >25 nutrition mentions	Cancer-related fatigue Survivorship
Patient-directed guidelines (39)	9 (23%) 1–10 nutrition mention 2 (5%) 11–25 nutrition mentions	Nasopharyngeal cancer Oral cancers Stomach cancer

* Complete count of nutrition mentions by guideline provided in Supplementary Materials.

**Table 4:** Key search terms for research review of malnutrition and nutrition-focused quality improvement programs (QIPs) and quality effectiveness process initiatives in cancer care.

String	Terms
Cancer	Cancer, neoplasm, tumor, oncology, carcinoma, sarcoma
Nutrition	Food, diet, nutrition
Care	Assessment, care plan, plan, counsel, council, diagnosis, consult, discharge education, education, evaluation, index, intervention, monitoring, oral nutrition supplement (ONS), screening, therapy, treatment
Efficacy	Efficacy, effectiveness, efficient, efficiency, effectiveness, effectivity
Quality	Improvement of quality, improvement of care, improvement of treatment, improvement of therapy

**Table 5:** Summary of abstracts identified through a research review of malnutrition and nutrition-focused quality improvement program (QIPs) and quality effectiveness process initiatives in cancer care.

Publication Type and Cancer Diagnosis	Title	Methodology	Conclusions
Article Head and neck cancer	Evaluating the effectiveness of a nutrition assistant role in a head and neck cancer clinic [42]	Evaluated the effectiveness of nutrition assistant performing screening/intervention in multidisciplinary head and neck clinic Provided training to nutrition assistants Compared outcomes between pre- and post-implementation of nutrition assistant role	Nutrition assistant roles resulted in improved patient satisfaction, maintenance of nutritional outcomes, and demonstrated effectiveness of role in supporting management of head and neck cancer patients within multidisciplinary treatment clinic
Conference abstract Neuroendocrine tumor (NET)	Evaluation of nutritional deficiencies in a new gastroenterology-led South Wales neuroendocrine tumor (NET) service [43]	Retrospective study with data collected from medical records of 99 patients who attended new gastroenterology-led service Compared data to 67 consecutive patients from previous service without gastroenterology input	Assessment addressing nutrition deficiencies was improved in new South Wales NET service incorporating gastroenterology Some assessments could be improved by increased dietitian involvement
Conference abstract Gastrointestinal (GI) cancer	A nutrition-focused quality improvement program to improve rate of documented nutrition plan at a safety-net hospital gastrointestinal (GI) oncology clinic [41]	Aimed to increase documented Registered Dietitian Nutritionist (RDN) nutrition assessment from 7% to 25% Arranged multidisciplinary sessions with healthcare team to identify barriers to nutritional interventions for GI oncology clinic patients Carried out Plan Do Study Act (PDSA) cycles as part of nutrition-focused QI program	Doubled rate of documented nutritional plan for Parkland Health and Hospital System GI cancer patients within month of starting second PDSA cycle intervention
Conference abstract Gastrointestinal (GI) cancer	Development of a nutrition-focused quality improvement program for new patients with cancer seen at the UTSW Simmons Comprehensive Cancer Center (SCCC) outpatient gastrointestinal (GI) oncology clinic [40]	Aimed to increase rate of documented clinical dietitian nutrition assessment to 65% within 90 days of new patient encounter Obtained baseline data from electronic medical record Arranged group sessions to apply quality improvement (QI) methodologies to determine steps to clinical dietitian documented nutritional plan Interviewed patient advocates to assess patient perspective Planned sequential PDSA cycles to improve rates of nutrition plan documentation; data obtained every 2 weeks	After first PDSA cycle, early 2-week assessment showed 28% documented rate of nutritional plan; this should increase with longer follow-up and subsequent PDSA cycles Malnutrition in GI cancer is prevalent and under-recognized in routine clinical encounters Addressing malnutrition is important from patient perspective
Conference abstract Not specified	Onconut^®^: Nutritional care optimization for cancer patients [44]	Aimed to observe if European Society for Clinical Nutrition and Metabolism (ESPEN) guidelines and best nutritional practices are followed Completed three consecutive 6-month descriptive studies: ○ Analysis of current practice compliance to ESPEN guidelines (OncoNut^®^ Day 1) ○ Best nutritional practices creation and implementation ○ Compliance evaluation after implementation (OncoNut^®^ Day 2)	OncoNut^®^ is successful experience of multidisciplinary care and has been well accepted Nutrition Risk Screening 2002 (NRS-2002) evaluation is more complicated than expected for non-nutritionists; thus, training actions are required to improve nutritional screening
Conference abstract Lung cancer	Evaluation of an evidence-based nutrition care pathway for lung cancer patients undergoing radiotherapy [45]	Aimed to evaluate compliance with each component of lung nutrition care pathway and make recommendations for improvement Conducted retrospective audit on 29 patients commencing radical radiotherapy Examined compliance with patient screening, timing of first contact, Patient-Generated Subjective Global Assessment (PG-SGA) completion, and post-treatment follow up	To improve compliance, feedback was provided to nutrition department and is to be presented to multidisciplinary team to improve awareness To increase completion of PG-SGA in final week, forms are now being attached to outpatient notes To improve follow-up post-treatment, dietitian reviews are recommended to be scheduled together with post radiotherapy medical review Ongoing monitoring and regular evaluation of the pathway is recommended
Conference abstract Not specified	Nutrition assistants and malnutrition in a cancer setting: Exploring an integrated model of care [46]	Aimed to evaluate effectiveness of nutrition assistant role within new malnutrition screening, assessment, and treatment model for inpatients Developed nutrition assistant position and competency training program Collected baseline data on adherence to model of care, malnutrition screening, and nutrition department activity and compared to post-implementation data	Nutrition assistant role can be effectively established in inpatient cancer setting Nutrition assistants were highly satisfied and confident in their role after completing in-house training program Results indicate that this role can assist in favorable patient outcomes and effective workforce planning

## Supplementary Materials

The following are available online at https://www.mdpi.com/2227-9032/8/3/227/s1. Table S1: NCCN Guidelines for Treatment of Cancer by Site, Table S2: NCCN Guidelines for Treatment of Cancer by Specific Patient Population, Table S3: NCCN Guidelines for Supportive Care, Table S4: NCCN Guidelines for Patients.
